# Structural Strength Analyses for Low Brass Filler Biomaterial with Anti-Trauma Effects in Articular Cartilage Scaffold Design

**DOI:** 10.3390/ma15134446

**Published:** 2022-06-24

**Authors:** Yan Yik Lim, Azizi Miskon, Ahmad Mujahid Ahmad Zaidi

**Affiliations:** 1 Faculty of Defence Science and Technology, National Defence University of Malaysia, Prime Camp, Sungai Besi, Kuala Lumpur 57000, Malaysia; myylim@gmail.com (Y.Y.L.); mujahid@upnm.edu.my (A.M.A.Z.); 2 Faculty of Engineering, National Defence University of Malaysia, Prime Camp, Sungai Besi, Kuala Lumpur 57000, Malaysia

**Keywords:** anti-trauma effects, metallic filler polymer reinforced, requisite strength, structural strength, morphology, superlattice structure

## Abstract

The existing harder biomaterial does not protect the tissue cells with blunt-force trauma effects, making it a poor choice for the articular cartilage scaffold design. Despite the traditional mechanical strengths, this study aims to discover alternative structural strengths for the scaffold supports. The metallic filler polymer reinforced method was used to fabricate the test specimen, either low brass (Cu_80_Zn_20_) or titanium dioxide filler, with composition weight percentages (wt.%) of 0, 2, 5, 15, and 30 in polyester urethane adhesive. The specimens were investigated for tensile, flexural, field emission scanning electron microscopy (FESEM), and X-ray diffraction (XRD) tests. The tensile and flexural test results increased with wt.%, but there were higher values for low brass filler specimens. The tensile strength curves were extended to discover an additional tensile strength occurring before 83% wt.%. The higher flexural stress was because of the Cu solvent and Zn solute substituting each other randomly. The FESEM micrograph showed a cubo-octahedron shaped structure that was similar to the AuCu_3_ structure class. The XRD pattern showed two prominent peaks of 2θ of 42.6° (110) and 49.7° (200) with *d*-spacings of 1.138 Å and 1.010 Å, respectively, that indicated the typical face-centred cubic superlattice structure with Cu and Zn atoms. Compared to the copper, zinc, and cart brass, the low brass indicated these superlattice structures had ordered–disordered transitional states. As a result, this additional strength was created by the superlattice structure and ordered–disordered transitional states. This innovative strength has the potential to develop into an anti-trauma biomaterial for osteoarthritic patients.

## 1. Introduction

Over the last few decades, researchers and scientists have attempted to fulfil the demands [1] of osteoarthritic patients [2,3] with an articular cartilage scaffold [4,5] equipped with requisite strength [6,7], cell growth factors [8,9,10], and anti-inflammatory properties [11,12]. Unfortunately, they invented biomaterials, such as hydrogels and their derivatives, that were equipped with the carriers of cell growth factors [13] and anti-inflammatory properties [14] but without the requisite strengths [15]. Finding a biomaterial with better mechanical properties does not seem to be a better choice. Therefore, the scaffold is a supportive structure [16,17] that has become a popular option to improve structural strength and structural integrity [18]. This biomimetic tissue engineering environment [19,20] was invented to treat articular cartilage tissue [21], microfracture [4,22], and osteochondral defects [23,24].

Some finite element and experimental research had been conducted on mechanical strengths, such as tensile and compressive strengths, for scaffold structural support [6,7]. Some in vivo, in vitro, and ex vivo studies [12,19,25,26] had been conducted on cell viability [27] based on the scaffold structure’s properties [23]. The harder and softer materials had lower and higher stresses, respectively [28], resulting in the failure of the softer material under higher stress [29]. This is happening in the harder scaffold biomaterial and the softer tissue cells, resulting in cell rupture. Therefore, this biomaterial is a poor choice for anti-trauma effects in the scaffold design because it does not protect the cells [30,31]. It is crucial to find alternative strengths, such as the structural strength for biomaterials in these scaffold applications.

In the present study, the softer low brass filler with a typical Mohs hardness scale of 5.5–6.0 [32] was used to replace the harder titanium dioxide filler [33] of 2.5–3.0 [34] in the existing reinforcement filler scaffold [35]. These test specimens were prepared by the metallic filler polymer reinforced fabrication method [36]. Tensile, flexural, field emission scanning electron microscopy (FESEM), and X-ray diffraction (XRD) tests were conducted to measure their mechanical and physical properties. Firstly, the biomaterials must provide the requisite mechanical strength for the scaffold. Secondly, the flexural strength was required to comply with the structural integrity requirement [16,26,37] for the scaffold’s design criteria [18,27]. Lastly, FESEM and XRD were used to study the physical characteristics of the microstructure. This study aims to discover alternative strengths and anti-trauma features for the scaffold design.

## 2. Materials and Methods

### 2.1. Fillers and Adhesive

The titanium dioxide filler with UV-Titan P 160 grade (AP&C, Boisbriand, QC, Canada) was an acicular crystal-shaped powder with a grain size diameter of 17 nm and a specific gravity density (ρ_t_) of 4.0 gcm^−3^ [38]. The low brass filler with the 80Cu20Zn grade (NovaScientific, Petaling Jaya, Malaysia) had a grain size diameter of 40 microns and a special gravity density (ρ_b_) of 8.6 gcm^−3^. The specific gravity density range of polyester urethane adhesive with the Loctite^®^ Frekote^TM^ grade (Henkel Adhesive Technologies, Düsseldorf, Germany) was 0.715 to 0.725 gcm^−3^.

### 2.2. Test Specimen Preparations

The preparations of test specimens for tensile and flexural tests are attached in Supplementary Materials, as shown in the supporting figure at the end. All the inner faces of the aluminium square mould were wrapped in aluminium foil to protect them from wear and tear. A 10-newton hand-press apparatus was made for the hot reinforcing process. Low brass and titanium dioxide powders were measured to mix each of them into the polyester urethane adhesive according to the filler composition weight percentages of 0, 2, 5, 15, and 30. The filler composition weight percentage was calculated using the weight fraction formula as shown in Equation (1):

(1) $$wt_{i}=\frac{w_{i}}{w_{T}}\times 100\%$$

where *wt_i_*, *w_i_*, and *w_T_* are the filler composition weight percentage, the filler weight, and the total weight of filler and polyester urethane adhesive, respectively.

For both tensile and flexural test specimens, their configurations complied with ASTM E8 [39]. However, the shapes of test specimens were simplified for easy processing. Their dimensions for the tensile and flexural tests were further characterised by International Standard ISO 527-2 as Tensile Testing for Plastics [40] and ISO 178 as Plastics-Determination of Flexural Properties [41], respectively.

### 2.3. Fabrication Method

**Figure 1:**
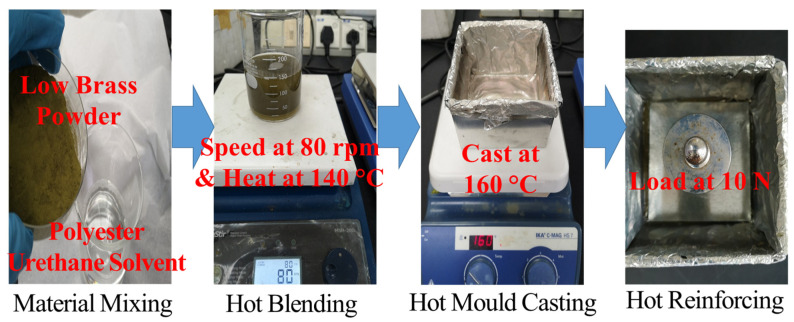
Metallic filler polymer reinforced process of biomaterial.

The fabrication method is a metallic filler polymer reinforced method [42] using two hot plate stirrers (Wisestir^®^, ProfiLab24 GmbH, Munich, Berlin, Germany) with the feedback control digital time function for blending and reinforcing with the aid of a 10-Newton hand-press apparatus. As shown in Figure 1, these processes included mixing raw materials and hot blending the mixture at 80 rpm speed at 140 °C for 6 min before pouring them into the mould casting with hot reinforcing at 160 °C for 6 min.

### 2.4. Tensile and Flexural Tests

For tensile tests, there were fifteen samples from each of the titanium dioxide and the low brass filler biomaterials, which consisted of three samples from each of the filler composition weight percentages, respectively. For flexural tests, there were fifteen samples produced, of which three were from each of the five low brass filler composition weight percentages. Both tensile and flexural tests were run at a constant speed rate of 3 mm min^−1^ by the Universal Testing Machine brand 5569 from Instron^®^ Norwood, MA, USA, which was equipped with a Dual Column Tabletop Universal Testing System with a maximum load of 50 kN. The tensile and flexural tests were configured with an additional 15% elongation extensometer and a one-point loading anvil, respectively.

### 2.5. Microscopy and X-ray Diffraction Tests

The low brass powder was investigated by FESEM brand Carl Zeiss^®^ GeminiSEM500 (Jena, Germany) and XRD instrumentation brand Smart APEX II, Bruker AXS GmbH (Karlsruhe, Germany). The FESEM used for morphology tests was controlled by the SmartSEM software and the GeminiSEM digital system control panel under a high vacuum mode of 2.97 × 10^−6^ mbar and an extra high voltage of 3000 eV. XRD instrumentat was used to identify the crystalline structures with Cu-Kα radiation of 1.5406 Å wavelength and Bragg’s scanning angle varied from 10° to 90° at ambient temperature [43].

## 3. Results Analyses of Additional Tensile Strength

### 3.1. Results Analysis of Tensile Strength and Strain at Break

**Figure 2:**
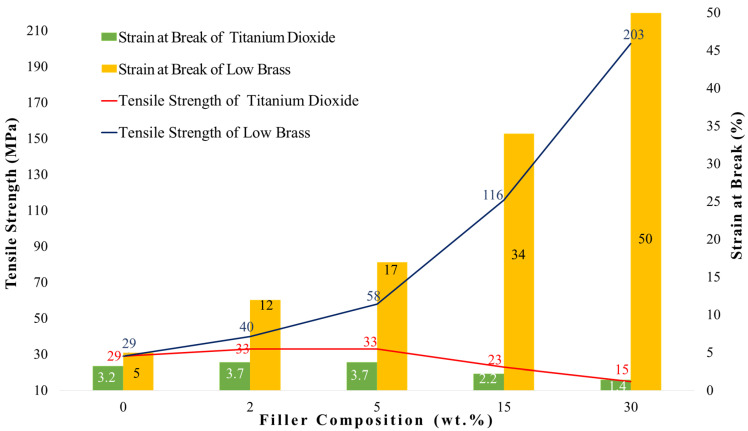
Tensile strength and strain at break for titanium dioxide and low brass fillers.

The tensile strength and strain at break results for titanium dioxide and low brass fillers were plotted against the composition percentages, such as 0%, 2%, 5%, 15%, and 30%, in biomaterials, as shown in Figure 2 [44]. The tensile strength and strain at break results for low brass filler increased exponentially with the filler composition percentages. However, the tensile strength and strain at break results of titanium dioxide filler increased until 2% filler composition and then decreased after 15% filler composition. These decrements were due to improper blending and fabrication methods [45] and weak oxygen [46] bonds in titanium dioxide. In addition, the tensile strength and strain at break results of low brass filler were higher than titanium dioxide filler for all similar filler composition percentages. As a result, low brass filler had higher mechanical strength than titanium dioxide filler for all percentages of biomaterials.

### 3.2. Determination of Requisite Tensile Strength for Articular Cartilage Scaffold

Traditionally, tensile strength was the main parameter used to measure the requisite strength for vascular supportive [47] structures for scaffold designs [18], as shown in Table 1. The tensile strengths of titanium dioxide and low brass were more than enough to maintain the structure’s support because they were four-times higher than tendon and ligament tissues. Therefore, the tensile strengths were over-strengthened for scaffold design. As shown in Table 1, low brass filler biomaterial had the requisite tensile strength (40–203 MPa) to replace the articular cartilage tissue (36–70 MPa) [48] for osteoarthritis therapy.

### 3.3. Curve Analysis of Tensile Strength Extension

**Figure 3:**
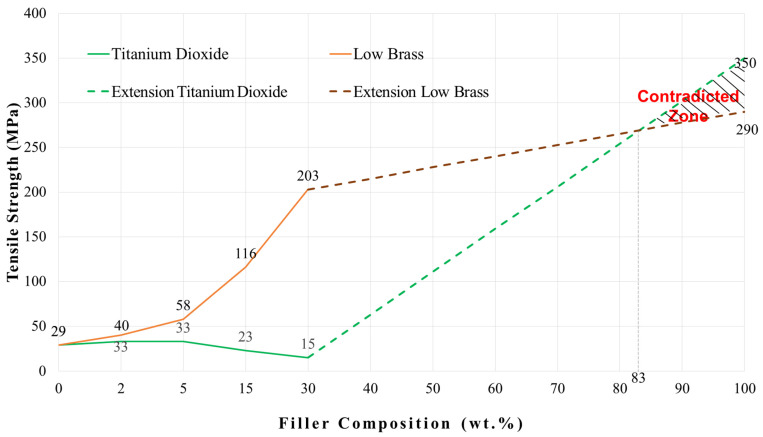
The contradicted zone between the extended curves of low brass and titanium dioxide filler tensile strengths.

The tensile strength curves in Figure 2 were extended to a 100% filler composition for low brass and titanium dioxide. The data of 100% filler compositions were taken from their pure materials, which were 350 MPa [33] and 290 MPa [49] for titanium dioxide and low brass, respectively. These data showed that titanium dioxide had a higher tensile strength than low brass, and this reversed the results of the low brass filler having a higher tensile strength than the titanium dioxide filler. Because of this reversed result, the extended curves were crossed and formed a contradicted zone, as shown by the black crossed lines in Figure 3. This contradicted zone described that the low brass filler obtained abnormally high tensile strength before 83 wt.% filler composition. This abnormally high tensile strength indicated that an additional tensile strength occurred during the fabrication process. As a result, the low brass filler biomaterial is better than titanium dioxide because of the additional tensile strength obtained during the fabrication process.

### 3.4. Mechanism Analysis of Additional Tensile Strength

**Scheme 1:**
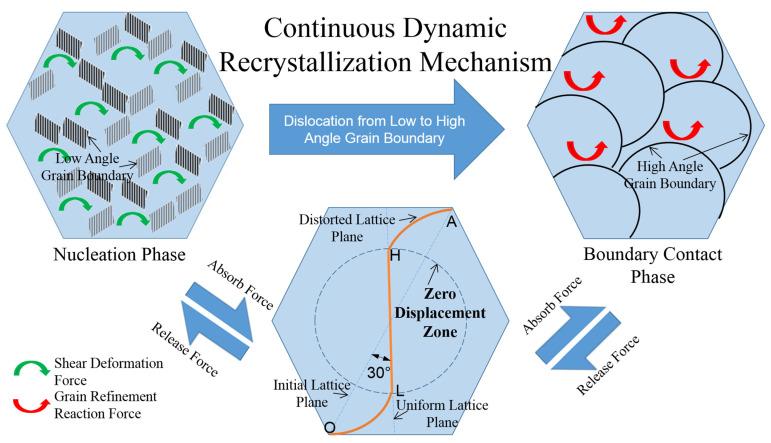
Continuous dynamic recrystallization mechanism in low brass with angle grain boundary dislocation, lattice plane distortion and forces.

The additional tensile strength was due to the continuous dynamic recrystallization mechanism consisting of shear deformation force and grain refinement reaction force [50], as shown in Scheme 1. The initial nucleation phase with the low angle grain boundary (AGB) was dislocated [51] by absorbing shear deformation forces into a boundary contact phase with high AGB [52] and then releasing the grain refinement reaction force to restore the initial phase [53]. The low brass filler in biomaterial sheared their AGB by increasing the strain at break, and tensile strength was increased by distorting the lattice plane [53], as illustrated by the OL and HA curves in Scheme 1. As a result of the increased tensile strength, the low brass filler biomaterial gained structural strength.

## 4. Results Analysis for Structural Integrity

### 4.1. Results Analysis of Flexural Stress, Flexural Displacement and Strain at Break

**Figure 4:**
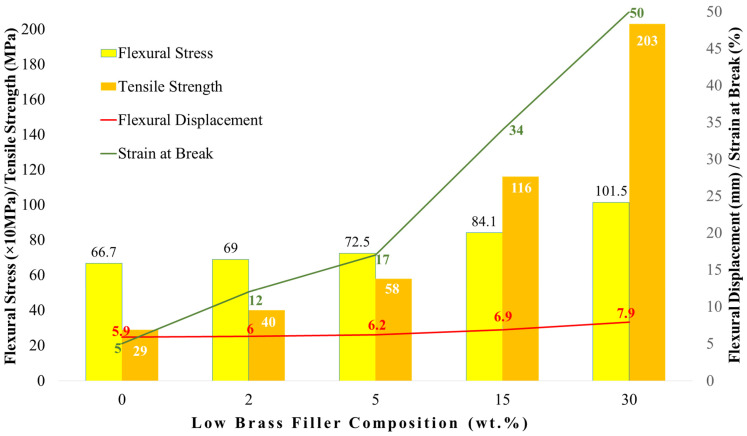
Flexural stress, flexural displacement, tensile strength, and strain at break against various low brass filler composition percentages.

The shear deformation and grain refinement reaction forces of the continuous dynamic recrystallization mechanism were investigated by flexural tests. The flexural stress and displacement results found compared to the tensile strength and strain at break results are shown in Figure 4. The higher flexural stress and displacement results showed that the low brass filler biomaterials had a higher ability to bend or rotate in their lattice planes. These higher flexural stress results were described by the shear deformation [54] and grain refinement reaction forces that occurred in Scheme 1. In addition, these higher flexural displacement results were described by the dislocation from low to high AGB, resulting in lattice plane distortion in Scheme 1. As a result, the higher flexural stress and displacement resulted from the continuous dynamic recrystallization mechanism, such as the shear deformation and grain refinement reaction forces, the AGB dislocations, and the lattice plane distortion.

The flexural stress and displacement results increased proportionally, but the tensile strength and strain at break results increased exponentially, with the low brass filler composition weight percentages, respectively. These results indicated that a small amount of low brass filler provided a higher flexural stress and displacement for the biomaterials. These results also indicated that a higher low brass filler composition percentage provided a higher exponential tensile strength and strain at break for the biomaterials. Therefore, a small amount of low brass filler was needed to provide shear deformation and grain refinement reaction forces, AGB dislocations, and lattice plane distortion [50,51,52,53]. As a result, a small amount of low brass filler provided the requisite strength in this scaffold design [6,7].

### 4.2. Forces and Displacements Analysis for Internal Structure Characteristics

**Scheme 2:**
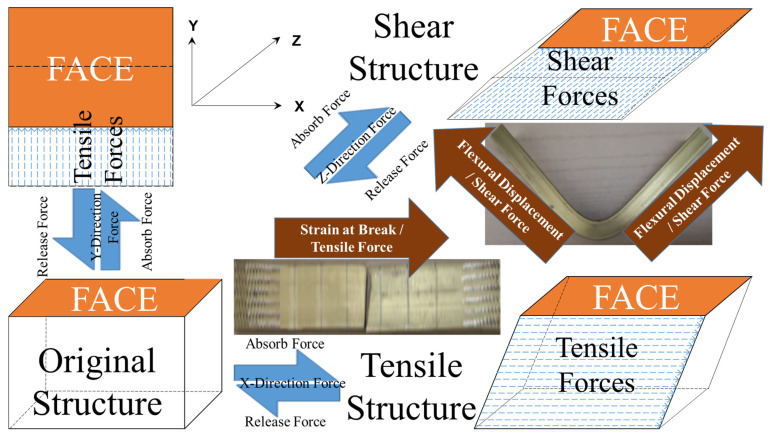
Internal structural characteristics of low brass filler biomaterial with the reversible mechanisms of absorbed and released forces.

To understand the continuous dynamic recrystallization mechanism that occurred, Scheme 2 was drawn to describe the X-, Y-, and Z-directions of the tensile, shear, and reaction forces, the AGB dislocations, and the lattice plane distortion. The original structure absorbed tensile forces to transform into the tensile structure, resulting in dislocation from the low AGB to the high AGB in the X-direction [55]. If the tensile stress was located under the elastic zone or before the strain at break, the tensile structure would return to the original structure by releasing forces, resulting in dislocation from the high AGB to the low AGB in the X-direction [56]. These reversible transformed structures and forces also occurred in the Y-direction. For the Z-direction, the original structure absorbed the shear or the shear deformation forces to transform into the shear structure, resulting in the rotation or bending or distortion of the lattice plane as shown in the middle right of Scheme 2. These shear forces were similar to those that roughened the interfaces in the interfacial energy dissipation study performed by Hao et al. [57]. If this structure retained structural integrity without failure, the shear structure would have returned to the original structure shear forces by releasing the shear or the grain refinement reaction forces in the Z-direction. As a result, these reversible tensile and shear structures involved absorbing and releasing forces using AGB dislocations and lattice plane distortion. These absorbed and released forces were similar to the interfacial dissipated and stored energies performed by Hao et al. [57]. This explains the exponential increment of flexural stress and displacement in Figure 4 because of the stacking fault energy being dissipated and stored temporarily in the interfacial between the tetrahedron and octahedron of the internal structure [58]. This discovery feature of a certain number of forces or energies being dissipated and stored was very important for articular cartilage scaffold designs. This is an anti-trauma effect feature that absorbs the forces or shocks from attacking the neocartilage tissue cells, preventing the cell rupture of osteoarthritis patients [30,31]. A numerical simulation study had been performed on this biomaterial to investigate the blunt-force trauma effect [59]. This addresses the too-hard problem of the scaffold’s biomaterial, which resulted in blunt-force trauma to the patient.

### 4.3. Determination of Structural Integrity for Articular Cartilage Scaffold Design

This articular cartilage scaffold was a supportive structure [16,17] requiring a structural integrity [18] feature in the biomimetic design of the tissue engineering environment [19,20]. As shown in Table 1, both titanium dioxide and the low brass filler biomaterials had the requisite tensile strengths to maintain the structural integrity of this supportive structure. The tensile and flexural test results showed higher tensile strengths and flexural stress, respectively, which provided the requisite mechanical strengths to maintain the structural integrity of the scaffold. In addition, the reversible continuous dynamic recrystallization mechanism absorbed and released the tensile and shear forces or energies using AGB dislocations and lattice plane distortion to dissipate and store them in the interfaced internal structure. This was a structural strength that allowed for higher flexural displacement without failure in order to maintain the structural integrity of the scaffold. However, this biomaterial will degrade sooner or later, resulting in a loss in tensile strength. This loss will eventually weaken the structural integrity of design [17,26,27,37]. Without structural integrity, the extracellular matrix will lose its function and the entire articular cartilage tissue network will malfunction. However, the degradation effect is not being studied on the structural strengths throughout this low brass filler biomaterial. Therefore, further experiments should be conducted to investigate the degradation effects on the structural integrity of the structure.

## 5. Discussion of Structural Characteristics and Reversible Mechanisms

### 5.1. Morphology Analysis

**Figure 5:**
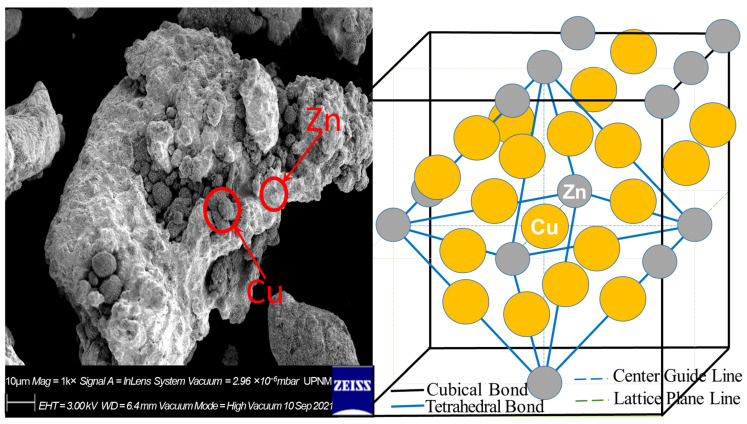
Morphology comparison between the low brass’s micrograph and the Cu-Zn superlattice structure class’s illustration.

The structural characteristics of biomaterial had been studied in our previous article [60]; thus, this low brass filler composition weight percentages in biomaterial was the focus of this study. An attempt was made to investigate the low brass filler’s role in these reversible continuous dynamic recrystallization mechanisms of biomaterials. Therefore, a micrograph was taken on low brass powder using a one-thousand magnification time FESEM test, as shown on the left of Figure 5. The appearance structure of this low brass micrograph result indicated that this belongs to the AuCu_3_ structure class (Cu_3_Zn), which is an alpha-brass family class. A simple unit cell illustration was drawn to describe this structure class with a three-dimensional solid cubo-octahedron shaped structure, as shown on the right of Figure 5.

Low brass is an alpha-brass family class that is metastable; thus, it can coexist with other family classes, such as beta- and gamma-brass [61,62]. That means that this unit cell may exist with more than two clusters, each comprising 26 atoms, as shown in Figure 5. Each cluster may constitute the vertices of the inner tetrahedron, outer tetrahedron, octahedron, and distorted cubo-octahedron shaped structure [61]. Therefore, the XRD tests were conducted to investigate these transitional-shaped structures.

### 5.2. Superlattice Structure Analysis

**Figure 6:**
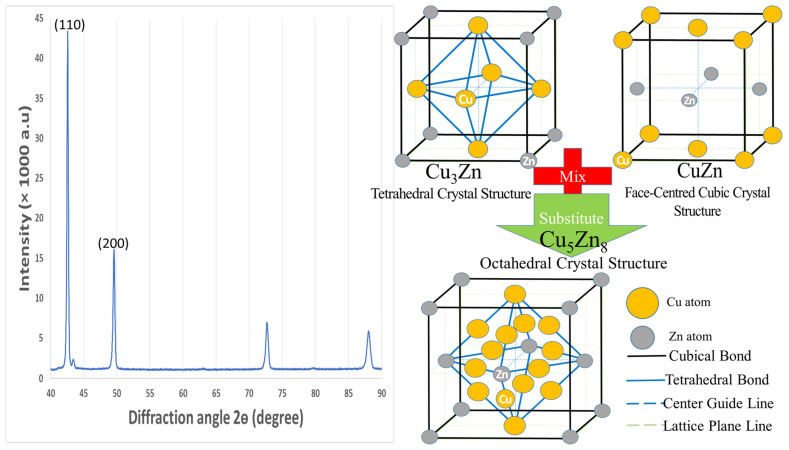
The X-ray diffraction pattern and the superlattice structure illustration for low brass powder.

The XRD pattern showed two prominent peaks (2pp) at the two theta diffraction angles (2θ) of 42.6° and 49.7°, which were Miller indices of (110) and (200), respectively, as shown at the left of Figure 6. This result indicated the typical face-centred cubic (fcc) crystalline structure with Cu and Zn atoms. The other XRD results also showed 2pp at 2θ of 43° and 50° for CuZn [63], Cu_3_Zn [64], and Cu_5_Zn_8_ [65]. Therefore, this is a superlattice structure, which is a combination of CuZn, Cu_3_Zn, and Cu_5_Zn_8_, as shown at the right of Figure 6. Referring to the FESEM result, this is a superlattice structure with a cubo-octahedron shape. This is happening because of the principles of fcc and 1:1 atomic composition ratio, which means either that one Cu at the centre or one Zn at the corner was substituted for each other. As a result, low brass was substituted by Cu and Zn with others in solid solutions to form a superlattice structure [62], as shown on the left of Figure 5.

The lattice spacing in lattice parameters was calculated using Bragg’s equation as shown in Equation (2):

(2) 2dsinθ=nλ

where *d*, θ, *n*, and λ denote lattice spacing, the angle between the wavevector incident plane and the lattice plane, an integer of the reflection order, and the wave length, respectively. The integer of the reflection order (*n*) and wave length (λ) were 1 and 1.5406 Å. The *d*-spacings in the lattice parameter were 1.138 Å and 1.010 Å for 2θ of 42.6° and 49.7°, respectively. The *d* of about 10 nm thickness indicated the typical thickness of the superlattice structure that was made up of alternative layers of two different materials [66]. As a result, the low brass structure is found to be appropriately described as a superlattice structure [67].

### 5.3. Discussion of Ordered-Disordered Transitional States of the Superlattice Structure

The two different materials of copper and zinc are grown into each other with a specific thickness in an alternative layer in the superlattice structure. The XRD pattern with 2pp of 2θ for copper, zinc, cart brass, and low brass was tabulated to investigate the substitution, as shown in Table 2. For copper powder, the 2pp of 2θ were similar for reference numbers 68 and 69, which were a little different from reference number 70, but their range difference was the same. For zinc powder, the 2pp of 2θ for reference numbers 71 and 72 were a little different, and their range difference was 4° and 8°. For the cart brass sheet (Cu_70_Zn_30_), the 2pp of 2θ for reference numbers 73 and 74 were a little different, and their range difference was the same. The XRD tests for copper, zinc, and cart brass were conducted in different external environments, so the little difference in degree was normal. For our finding low brass (Cu_80_Zn_20_), the 2pp of 2θ were a little different from all the 2pp of 2θ for copper, zinc, and cart brass, but the range difference was about 7°, which was similar to them. As a result, all these 2pp of 2θ in Table 2 indicated that the superlattice structure is the ordered–disordered transitional states.

**Scheme 3:**
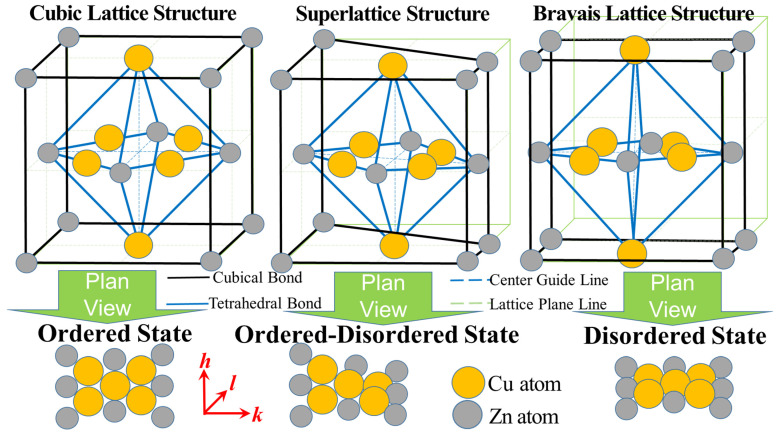
An illustration of ordered–disordered transitional states of the Cu_3_Zn superlattice structure class using atoms, lattice planes, and the *hkl* Miller indices.

This ordered–disordered transitional state of the superlattice structure was created during the fabrication of biomaterial made of low brass and polyester urethane. This biomaterial solution was heated at elevated temperatures of up to 160 °C and then slowly cooled in a random substitution of solvent and solute atoms to a solid solution with their preferred positions [75]. Below the critical temperature, this Cu_3_Zn superlattice structure was transformed between a symmetrical and an asymmetrical structure by using the open circle of Zn atoms to occupy other atomic sites [76]. This parent fcc superlattice structure with Miller indices *h, k,* and *l* was derived into structures including equivalent original cubic, unequivalent intersticing species, and reducing *l*-axial length atomic sites [77], as shown on the left, middle, and right of Scheme 3, respectively. This Cu_3_Zn superlattice structure may be stronger in an intermediate ordered–disordered state compared to those in fully ordered and disordered states at lower and higher temperatures, respectively [78].

As shown on the left of Scheme 3, the Cu_3_Zn alloy with the parent fcc lattice structure, where an atom set ordered for Cu at the face center and Zn at the cube vertices occupied the octahedral structure. These Zn atoms occupied the cube vertices or corners of the fcc lattice structure or solid solution sites, which may be preferentially configured with another atom to form a cluster plus glue atom ([Zn-Cu_12_]Zn_6_) [79] in which twelve Cu atoms are occupied by six Zn atoms [80]. This configuration induced dislocations of atoms, resulting in the plasticity state that bore directly on the Bravais lattice structure of important alloy systems [78], as illustrated on the right of Scheme 3. However, this intrinsic dislocation was restricted by the sessile (immobile) dislocation theory [81], whereby shape changes generally demanded several independent (dislocation) slip systems [78]. These systems were operated with limited slip and ductility modes, which might result in the freedom restriction of inhibited slip during the plasticity deformation in a polycrystalline matrix [81]. As a result, the abovementioned slip and anti-slip caused the tetragonal distortions of the parent fcc lattice structure and lowered the cubic symmetry, as illustrated in the middle of Scheme 3 [77].

### 5.4. Discussion of Spatial Configuration Arrangements and Lattice Plane Distortions of the Cu_3_Zn Superlattice Structure Class

**Scheme 4:**
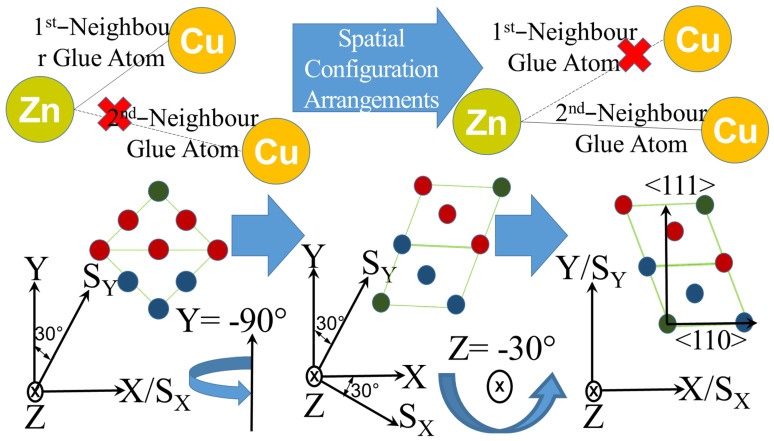
Illustration of spatial configuration arrangements (**top**) and the evolution of lattice plane distortion by the shear–strain axis rotation and three-colour sphere balls in crystal structure (**bottom**).

The Zn atoms in the open circle attempted to configure the Cu_3_Zn ordered state with one of the possible low-temperature nearest neighbor configurations [79]. This was similar to the dislocation from the ordered state at a higher temperature to the disordered state at a lower temperature by occupying another atomic site. However, this dislocation was metastable and restricted by several independent slip systems and ductility modes from the sessile dislocation theory, as discussed above [81]. The spatial configuration arrangements were used to better describe the transformation between ordered and disordered states [79]. This was a dissimilar inter-atomic bonding tendency between the Zn solute and the Cu solvent to occupy the space of the cluster, resulting in gaining relatively high stable structures or special solid solutions [80]. An illustrated schematic was drawn to describe the phenomena of this Zn atom occupying the cluster with either the first-nearest or second-nearest neighbor of the Cu atom, forming a cluster plus glue atom [Zn-Cu_12_]Zn_6_, as shown at the top of Scheme 4. The Zn reactant molecules occupied the cluster, involving chemical substance bonding energies in the short range order [82]. Therefore, the additional tensile strength found in Figure 3 was the spatial configuration bonding strength of the low brass filler biomaterial [83]. As a result, these energies were absorbed and released during the bonding and debonding chemical processes in biomaterial substances.

The continuous dynamic recrystallization mechanisms as mentioned in Section 3.4 were further analysed by their microstructural characteristics in the evolution of the lattice plane. The shear deformation and the grain refinement reaction forces grew the dislocations and restricted the slip systems, respectively, resulting in the distortion in the lattice plane’s evolution. An illustration was drawn to depict this lattice plane distortion evolving in a single-crystal structure but ending with the same axis direction as shown at the bottom of Scheme 4. The results showed that the final orientations of all axes were the same after the internal microstructural characteristics in texture were evolved by shear–strain forces from Y-axial and Z-axial [84]. Firstly, the nine three-colour sphere balls were in a square-shaped lattice plane with the axis shearing from Y-axial to S_Y_-axial at 30°. Secondly, the axis was rotated by −90° at Y-axial in order to shear 30° from X-axial to S_X_-axial, resulting in eight sphere balls in two prism-shaped lattice planes, as shown in the middle of Scheme 4. Lastly, the axis was rotated by −30° at Z-axial in order to shear −30° from S_X_-axial and S_Y_-axial to X-axial and Y-axial, respectively, resulting in eight sphere balls in the reverse direction of the two prism-shaped lattice planes. After these three rotations, the shear–strain axes had the same directions, but the lattice planes were rotated and distorted [85]. As a result, these lattice plane distortion analyses showed that the low brass filler biomaterial had the structural integrity and ability to bend without any deformation of the axes directions.

This spatial configuration arrangement increased the lattice plane distortions but was restricted by the “out-of-phase” or defect interfaces on either boundary side of the structure, known as the anti-phase boundary (APB) [86]. This phase also began to dislocate into the disordered state or matrix that was stacking fault energies needed to break the strong anisotropy deformation [81]. This energy per unit area of interface was referred to as APB energy, γ*_APB_*, and it could be isotropic or anisotropic [78,86]. This APB energy was calculated based on the energy differences solely due to the defect between the APB and the non-APB pristine atomic structures [87]. The APB energy was the defect formation energy per unit area and was expressed in Equation (3) [87]:

(3) $$\gamma_{APB}=\frac{E_{APB-}E_{pris}}{A}$$

where *E_APB_*, *E_pris_*, and *A* are the total energy of the APB atomic structure, pristine atomic structure, and area, respectively. The relationship between this total energy of the APB atomic structure and the total residual membrane stress of 650 MPa found in my previous article should be further investigated [59]. As a result, this contributes to the understanding of APB, membrane failure, stacking fault, and heterophase interface energies.

## 6. Conclusions

The tensile strength and strain at break for titanium dioxide filler and low brass filler increased with the filler composition percentages. The tensile strength curves of low brass and titanium dioxide were extended to a 100% filler composition to find a contradicted zone after an 83% filler composition was successfully completed. This contradiction was due to the tensile strength of the titanium dioxide pure material being higher than that of low brass, but its filler was lower reversely. This was because of a new structural strength found in low brass filler biomaterial during the metallic filler polymer-reinforced process. This structural strength was created by the continuous dynamic recrystallization mechanism, including the shear deformation force and grain refinement reaction force. In addition, flexural stress and flexural displacement increased with the low brass filler. The flexural stress and displacement results increased proportionally, but the tensile strength and strain at break results increased exponentially, with low brass filler composition weight percentages, respectively. This result indicated that only a small amount of low brass filler provided the requisite mechanical strength and the structural integrity of the structure successfully. This provides an important guideline for future scaffold design. The absorbing and releasing forces created by the AGB dislocations and the lattice plane distortions resulted in higher flexural stress and displacement. Through this mechanism, the energy was being dissipated and stored temporarily in the reversible tensile and shear structures, resulting in an anti-trauma feature for the patient. This innovative feature has an external shock prevention potential to be developed for the articular cartilage scaffold.

The FESEM investigation found that the low brass microstructure had a similar appearance to the AuCu_3_ structure class (Cu_3_Zn) with a solid cubo-octahedron shaped structure. Because low brass is a metastable alpha-brass family class, it can coexist in solid solutions with a multi-shaped superlattice structure with others, such as beta- and gamma-brass. The XRD pattern had 2pp at the 2θ of 42.6° and 49.7°, indicating the typical fcc crystalline structure of Cu and Zn atoms with Miller indices of (110) and (200), respectively. While compared with the other XRD results of CuZn, Cu_3_Zn and Cu_5_Zn_8_ also had 2pp at the 2θ of 43° and 50°; this meant that low brass was substituted for each other in solid solutions to form a superlattice structure. The *d*-spacing in the lattice parameter was calculated using Bragg’s equation with an *n* of 1 and λ of 1.5406 Å for the 2θ of 42.6° and 49.7° to obtain 1.138 Å and 1.010 Å, respectively. The superlattice structure was further verified by this *d* of about 10 nm thickness. This XRD pattern with the 2pp of 2θ was used to compare with other results of copper, zinc, and cart brass that indicated this was a superlattice structure with the ordered–disordered transitional states. This superlattice structure substituted solvent and solute atoms randomly to transform between the ordered state and the disordered state. The intermediate state could be the stronger state by reducing the symmetrical structure using the *l*-Miller index accordingly. The Zn atom at the cube vertices of the fcc lattice structure was configured preferentially in a plasticity state to form a cluster plus glue atom ([Zn-Cu_12_]Zn_6_) with a Bravais lattice structure. However, these intrinsic dislocation systems were restricted by limited freedom, inhibited slip, and low ductility modes, resulting in tetragonal distortions in a polycrystalline matrix.

The spatial configuration arrangement was Zn atom configured with its nearest neighbor to have a lower possible temperature, which was similar to the dislocation from the high-temperature ordered state to the low-temperature disordered state. This cluster occupation involved chemical substance bonding energies to gain a relatively stable structure. The internal microstructural characteristics in texture were rotated by −90° from Y-axial and −30° from Z-axial to describe the distorted lattice planes, but shear–strain axes had the same directions at the end. This lattice plane distorted without any axial deformation, which showed the structural integrity of the low brass filler biomaterials with bending ability. This spatial configuration arrangement increased lattice plane distortions but was restricted by the APB interfaces on either boundary side of the structure. APB energy included stacking fault energy, which was needed to break strong anisotropy deformation into the disordered state. However, the relationship between the APB total energy and total residual membrane stress should be further investigated and clarified. Therefore, further experiments gained more understanding of APB, membrane failure, stacking fault, and heterophase interface energies.

## Figures and Tables

**Table 1:** Tensile Strengths of Titanium Dioxide, Low Brass, Ligament, and Tendon Tissues.

Property	Units	Titanium Dioxide	Low Brass	Ligament Tissue	Tendon Tissue
Tensile Strength	MPa	350 ^a^	290 ^b^	36 ^c^	70 ^c^

Note: the superscripts of a, b, and c were the references to [33,48,49], respectively.

**Table 2:** Two prominent peaks of X-ray diffraction pattern for copper, zinc, cart brass, and low brass.

Materials	Diffraction Angle 2θ (°)		References
Copper powder (Cu)	43.3	50.3	[68,69]
	50.7	59.3	[70]
Zinc powder (Zn)	39.5	43.7	[71]
	42.1	50.8	[72]
Cart brass sheet (Cu_70_Zn_30_)	40.9	47.7	[73]
	42.3	49.6	[74]
Low brass (Cu_80_Zn_20_)	42.6	49.7	

## Data Availability

Not applicable.

## Supplementary Materials

The following supporting information can be downloaded at: https://www.mdpi.com/article/10.3390/ma15134446/s1, Supporting figure attached as in Supplementary Materials. Figure S1: Preparations of test specimens.
