# Supplementary figures and images for: Structural Strength Analyses for Low Brass Filler Biomaterial with Anti-Trauma Effects in Articular Cartilage Scaffold Design
Source: Materials (Basel). 2022 Jun 24;15(13):4446. doi: 10.3390/ma15134446 (PMC9267688; doi:10.3390/ma15134446)

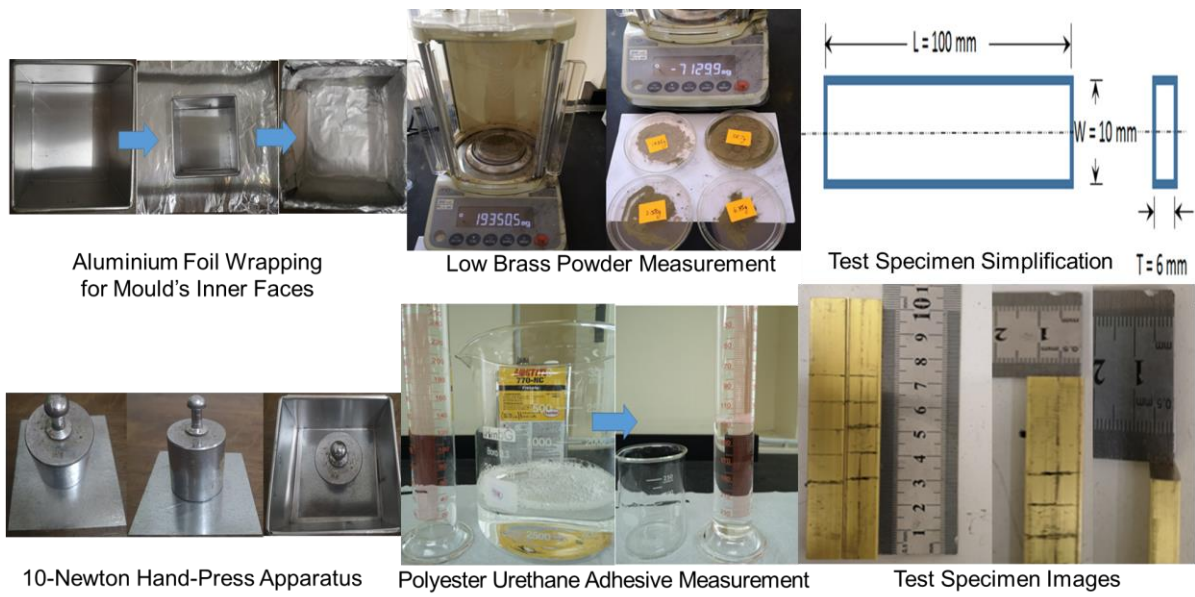

**Figure S1.** Preparations of test specimens.

Supplement: Supplementary file 1 [file materials-15-04446-s001.zip › materials-1695950-supplementary.pdf]
